# Use of the National Cancer Institute Patient-Reported Outcomes version of the Common Terminology Criteria for Adverse Events to assess treatment tolerability in pulmonary arterial hypertension: qualitative patient research findings in current and former users of oral selexipag

**DOI:** 10.1186/s41687-023-00673-w

**Published:** 2023-12-18

**Authors:** Stacy Davis, Teresa Edwards, Lindsey Norcross, Sheri Fehnel, Amélie Beaudet, Marie Eckart, John Fastenau

**Affiliations:** 1 Janssen Global Services, LLC, Horsham, PA USA; 2 RTI Health Solutions, Research Triangle Park, NC USA; 3 Actelion Pharmaceuticals Ltd, a Janssen Pharmaceutical Company of Johnson & Johnson, Allschwil, Switzerland

**Keywords:** Adverse events, Patient-reported outcomes, PRO-CTCAE, Pulmonary arterial hypertension, Selexipag, Side effects, Tolerability

## Abstract

**Background:**

Understanding patients’ perspectives regarding drug tolerability, in addition to effectiveness, provides a complete picture of the patient experience and supports more informed therapeutic decision-making. The item library of the National Cancer Institute’s Patient-Reported Outcomes version of the Common Terminology Criteria for Adverse Events (PRO-CTCAE) was developed to measure patient-reported frequency, severity, and interference of adverse events (AEs) associated with cancer therapies. This qualitative interview study assessed the suitability of items selected from the PRO-CTCAE library for assessing tolerability of selexipag, a medication targeting the prostacyclin pathway for patients with pulmonary arterial hypertension (PAH).

**Methods:**

Two rounds of 10 qualitative, web-assisted telephone interviews following a semi-structured guide were conducted in individuals with recent experience taking oral selexipag for PAH. Each interview included concept elicitation to gather participants’ perspectives on symptomatic AEs (type, frequency, severity, and interference) and cognitive debriefing of PRO-CTCAE items addressing the most frequently reported AEs of oral selexipag.

**Results:**

Interviews were conducted with 20 participants with PAH (mean [range] age 50 [24–68] years; 75% female; 85% in World Health Organization Functional Class II–III), comprising different races/ethnicities, levels of education, and employment status. Fifteen participants were currently treated with selexipag; five had taken selexipag for ≥ 6 months before discontinuing. The most frequently reported AEs included headache, jaw pain, and nausea (n = 15, 12, and 10 participants, respectively). Diarrhea and headache were identified as the most bothersome AEs by 5 and 4 participants, respectively. Some AEs were transitory (e.g., jaw pain); others were long-lasting (e.g., muscle pain). Based on findings from Round 1 interviews, a flushing item was added and the PRO-CTCAE general pain item was modified to be specific to jaw pain for testing in Round 2. Interview findings identified the following AEs as relevant to assess in a PAH clinical trial: nausea, vomiting, diarrhea, flushing, jaw pain, headache, aching muscles, and aching joints.

**Conclusions:**

The PRO-CTCAE items selected in this study and the additional symptomatic AEs identified as patient-relevant have the potential to be included in assessments capturing the patient perspective on tolerability in future studies of selexipag and possibly other PAH therapies.

**Supplementary Information:**

The online version contains supplementary material available at 10.1186/s41687-023-00673-w.

## Background

Traditionally, safety of a medication has been reported in publications and product labels in terms of clinician-reported incidence and severity of adverse events (AEs) and related discontinuations over the course of an interventional clinical trial. However, this does not provide information on how patients themselves perceive their burden [1]. Tolerability, a separate concept from safety, refers to the patient perspective on adverse drug reactions [2]. It is important to assess not only the safety profile of a medication but also its tolerability, because the latter is a driver of patients’ long-term treatment adherence and, ultimately, the success or failure of the drug [2]. To provide a more complete picture of the patient experience and support informed therapeutic decision-making, assessment of tolerability should incorporate direct measurement of the patient’s feelings and functioning [3].

There is increasing recognition of the importance of incorporating the patient perspective in the processes of drug development and evaluation [4–6], and in the past decade the approvals of many new drugs by the US Food and Drug Administration (FDA) and the European Medicines Agency (EMA) have been informed by the results of patient-reported outcomes (PRO) assessments [7, 8]. To capture the patient perspective on the tolerability of cancer therapies, the National Cancer Institute (NCI) developed a PRO version of the Common Terminology Criteria for Adverse Events (CTCAE) [9–11]. The PRO-CTCAE library comprises 124 items that measure the frequency, severity, and interference with daily life (as appropriate) of 78 symptomatic AEs [12]. The library and interview form builders are publicly available online from the NCI [13]. Qualitative research supports the content validity of items in the PRO-CTCAE library [14–16] and their response scales [17] in the context of cancer treatment.

Although the PRO-CTCAE was developed for cancer trials, its extensive item library could make it a useful tool for assessing treatment tolerability outside of oncology [12, 18]. To date, literature that reports efforts to evaluate the applicability of the PRO-CTCAE in other diseases has been sparse. Hazlewood et al. (2022) conducted an exploratory online survey of patients with rheumatoid arthritis (RA), which found that respondents attributed many AEs in the PRO-CTCAE library to their RA medications [19]. Hughes et al. (2022) described the protocol for a study to assess the feasibility of using an electronic PRO system, including the PRO-CTCAE and other instruments, in trials of advanced therapies for autoimmune disorders [20]. Craig and Mitchell (2016) developed a checklist for the measurement of menopausal symptoms by integrating items from the PRO-CTCAE [21].

No published studies have reported the use of the PRO-CTCAE in pulmonary hypertension (PH), a heterogeneous set of disorders characterized by elevated pulmonary arterial pressure [22]. Improving the measurement of tolerability would be of value in clinical studies of treatments for the PH subgroup pulmonary arterial hypertension (PAH), a rare, progressive, chronic disease of the pulmonary vasculature associated with debilitating symptoms, including breathlessness, fatigue, weakness, chest pain, lightheadedness, fainting, abdominal distension, and swelling of the legs and ankles [23, 24].

Several PAH-specific drugs are currently approved for treatment of PAH disease, each of which targets dysregulation in one of three key pathways involved in the pathophysiology of PAH—the prostacyclin, nitric oxide, and endothelin pathways [22, 23]. All PAH-specific therapies have characteristic AEs attributable to their mechanism of action [25], and AEs associated with medications targeting the prostacyclin pathway include headache, diarrhea, flu-like symptoms, jaw pain, muscle spasm, flushing, and nausea [26]. For medications targeting the prostacyclin pathway, the effective dose differs among patients, requiring careful dose titration for each individual to maximize effectiveness while minimizing AEs and optimizing tolerability [27, 28]. For example, identifying each patient’s optimal dose of the oral selective IP prostacyclin receptor agonist selexipag requires individualized up-titration to the highest tolerated dose, which can range from 200 µg twice daily to 1600 µg twice daily; the maintenance dose is the highest tolerated dose reached during dose titration [28–30]. In addition to improving tolerability at the individual level, the development of new PAH-specific therapies (or alternative formulations of existing therapies) with more favorable safety and tolerability profiles for use within this patient population could address an important unmet medical need.

To facilitate evaluations of tolerability in future PAH clinical trials, a measurement strategy that addresses symptomatic AEs of importance to patients is needed. The objective of this study was to gather insights directly from patients to better understand their experiences, perceptions, and prioritization of tolerability concerns, including an evaluation of the applicability of the PRO-CTCAE item library in PAH. Specifically, qualitative interviews were conducted in patients with PAH and prior experience with selexipag to inform the development of a PRO measurement strategy for implementation in clinical trials evaluating and comparing the tolerability of selexipag and potentially other medications targeting the prostacyclin pathway.

## Methods

### Study design

Qualitative, in-depth, cross sectional, web-assisted telephone interviews were conducted with individuals who had recent experience taking oral selexipag as a treatment for PAH. All interviews included two components: (1) concept elicitation to gather data on patients’ experiences of selexipag treatment, including likes, dislikes, and experiences during the titration and maintenance periods as well as detailed information on the frequency, severity, and interference from symptomatic AEs; and (2) cognitive debriefing to obtain participant feedback regarding the content validity of selected PRO-CTCAE items for assessing tolerability issues associated with selexipag.

The present study was reviewed by RTI International’s institutional review board and determined to be exempt.

### Participants

Interview participants were identified and recruited by Rare Patient Voice (Towson, MD, USA), a patient recruitment group for qualitative research, with a focus on specialty patient populations. Using an in-house patient database, Rare Patient Voice identified potentially eligible participants. Individuals who expressed interest in participating in the study were screened for eligibility and those who qualified were scheduled for interviews. A sample size of 20 was planned based on previous research indicating that concept saturation can be reached in as few as 10–12 interviews [31–33].

The following eligibility criteria were applied: age 18–79 years; self-reported clinical diagnosis of PAH confirmed via right heart catheterization (a requirement for a definitive diagnosis of PAH [34]); currently taking selexipag or stopped taking selexipag within the past 12 months; US resident; able to read, speak, and understand English and provide informed consent; and willingness to participate in a 60-minute, audio-recorded, web-based interview. Including participants who had recently discontinued selexipag ensured that important views on the tolerability of the drug were captured, given that the titration process is based on tolerability and therefore can be challenging, leading to some patients stopping selexipag therapy during this period [35].

Demographic data captured during screening included age, gender, race and ethnicity, and highest level of education attained. Clinical information captured during screening included a self-reported measure of World Health Organization (WHO) functional class (FC) [36] (a four-level scale commonly used in PAH to assess disease severity based on activity limitation [22, 37]) and patient-reported global rating of PAH symptom severity (none, mild, moderate, and severe). Demographic diversity was targeted to ensure a participant population that was reasonably representative of the PAH population in the US.

### Interviews

Each interview was conducted over Zoom by two members of the research team (TE and LN) with extensive qualitative research experience; one researcher led the discussion, while the other researcher took field notes. Each interview was audio recorded, transcribed to facilitate analysis, and deidentified (i.e., any information that could possibly identify a patient was removed from the transcripts).

All interviews followed a semi-structured guide to ensure that data were collected in a systematic and consistent manner. The interview guide was designed to meet study objectives while also encouraging spontaneity of responses and a conversational tone throughout the interviews. Before the start of each interview, the interviewer explained the purpose of the study and interview procedures, and obtained verbal consent for the interview and audio recording.

Interviews were conducted in two rounds of 10 interviews each, and both rounds included concept elicitation and cognitive debriefing components.

#### Concept elicitation

Each interview began with open-ended questions asking participants to describe their experiences with PAH, such as when they were diagnosed and their treatment history. These general questions were followed by more targeted questions about participants’ experiences when taking selexipag.

Interview participants were asked to describe all symptomatic AEs they experienced when taking selexipag, along with the frequency and severity of each side effect, the stage of treatment (i.e., titration or maintenance) at which it was experienced, any changes in either frequency or severity that they experienced over time, and whether and how the side effect interfered with their daily life. Participants were also asked, “Thinking about any negative effects that you experienced, which were the most bothersome to you? Why did these bother you the most?” If not spontaneously mentioned, interviewers queried participants about known AEs of selexipag reported in its pivotal phase 3 trial, GRIPHON [28], that are already included in the PRO-CTCAE item library, including headache, nausea, back pain, muscle pain, diarrhea, vomiting, and joint pain. In addition, the following AEs reported in GRIPHON but not included in the PRO-CTCAE library were probed: nasopharyngitis (cold), flushing (blushing, blotchiness), pain in the extremities, and jaw pain.

#### Cognitive debriefing

After the concept elicitation portion of the interview, participants were cognitively debriefed on selected items from the PRO-CTCAE item library, providing feedback on the relevance of the concept in a clinical trial for PAH, the appropriateness of the frequency, severity, and interference questions, and the appropriateness and feasibility of the 7-day recall period recommended by the NCI [10]. Participants’ opinions were also solicited about the appropriate frequency for collecting these data from clinical trial participants in the titration and maintenance phases.

Items were selected from the PRO-CTCAE library for debriefing based on the most frequent clinician-reported AEs in the GRIPHON trial that were attributable to selexipag (rather than being symptoms associated with PAH) and reported at a higher incidence than with placebo [28].

Items debriefed in the Round 1 interviews addressed the severity, frequency, and interference (as available within the PRO-CTCAE library) of nausea, vomiting, loose or watery stools (diarrhea), cough, pain (i.e., general pain), headache, aching muscles, and aching joints. After completion of the first 10 interviews (Round 1), revisions were made based on participant feedback to ensure collection of the most salient concepts. Specifically, based on findings from Round 1, in the final 10 interviews (Round 2) the PRO-CTCAE general pain item was modified to ask specifically about jaw pain and an interference item was added for diarrhea. In addition, the PRO-CTCAE item about cough was removed from the interview guide for Round 2 and an item about “blushing” was added, along with probes to elicit the best terminology for this concept (“blushing”, “flushing”, or “blotchiness”).

### Data analysis

To ensure consistency, all coding and analysis was conducted by the same two RTI Health Solutions personnel who conducted the interviews. Initial identification of themes began as interviewers debriefed following each interview and noted important concepts and dominant trends that were emerging. When all interviews were complete, thematic analysis methods were used to analyze the interview data [38]. A prespecified coding framework was applied, and the framework was expanded during coding to be comprehensive of all relevant concepts that emerged from the interviews, allowing for assessment of patterns in participants’ responses [45].

As this qualitative study was designed to generate insights rather than to test *a priori* hypotheses, no tests of statistical significance were performed. Study findings are reported using summary statistics and direct patient quotations.

## Results

### Participant characteristics

A total of 20 adults with PAH were interviewed between 17 May and 15 June 2022. As presented in Table 1, participants ranged in age from 24 to 68 years (mean 50 years), were predominantly (75%) female, comprised different races/ethnicities, and were diverse in level of education and employment status.

**Table 1:** Interview participant characteristics

Characteristic	Total sample (N = 20)
Age (years), mean (SD), range	50 (11), 24–68
Gender, n (%)	
Female	15 (75)
Male	5 (25)
Race/ethnicity,* n (%)	
White	15 (75)
African American or Black	3 (15)
Asian	1 (5)
Hispanic, Latin American, or Latinx	1 (5)
Education, n (%)	
Less than high school	1 (5)
High school diploma or equivalent (e.g., GED)	4 (20)
Technical school or associate’s degree	3 (15)
Some college	4 (20)
College degree (bachelor’s degree)	6 (30)
Professional or advanced degree	2 (10)
Employment status, n (%)	
Full time	6 (30)
Part time	3 (15)
Self employed	2 (10)
Unemployed and not looking for work	5 (25)
Retired	4 (20)
Currently taking selexipag, n (%)	
Yes	15 (75)
No longer taking	5 (25)
WHO Functional Class,^ †^ n (%)	
I	2 (10)
II	10 (50)
III	7 (35)
IV	1 (5)
Severity of current PAH symptoms (self-report), n (%)	
Mild	6 (30)
Moderate	12 (60)
Severe	2 (10)
PAH diagnosis year, mean (SD), range	2015 (4.6), 2004–2021
Current PAH treatment regimen, n (%)	
Selexipag monotherapy	0
Selexipag and a medication targeting one other pathway (double therapy)	3 (15)
Selexipag and medications targeting two other pathways (triple therapy)	12 (60)
Not currently taking selexipag	5 (25)
Current PAH medication(s),*^‡^ n (%)	
Nitric oxide pathway	
Tadalafil	8 (40)
Sildenafil	7 (35)
Riociguat	5 (25)
Endothelin receptor antagonists	
Macitentan	10 (50)
Ambrisentan	5 (25)
Bosentan	1 (5)
Drugs acting on the prostacyclin pathway	
Selexipag	15 (75)
Treprostinil (intravenous or subcutaneous)	3 (15)
Treprostinil (inhaled)	2 (10)

GED: General Educational Development; PAH: pulmonary arterial hypertension; SD: standard deviation; WHO: World Health Organization

*Percentages may not sum to 100% because participants may select more than one. All drugs are oral unless otherwise specified

^†^Self-reported using the Pulmonary Hypertension Functional Classification Self-Report, © 2021 United Therapeutics Corporation [36], which was adapted with permission from the Pulmonary Hypertension WHO Functional Classification System

^‡^In addition to PAH-specific medications, 6 participants volunteered that they currently take a diuretic, and 1 participant each volunteered that they take the following supportive therapies: rivaroxaban, bumetanide, potassium chloride, low-dose aspirin, statin, calcium channel blocker, oxygen as needed

Most participants (85%) were in WHO FC II–III according to self-report, and 18 (90%) self-reported their PAH severity as mild or moderate (Table 1). On average, patients had been diagnosed with PAH 4.6 years prior to the interview, but time since diagnosis varied widely, from 1 to 18 years.

Nearly all participants currently taking oral selexipag (14 of 15; 93%) were taking two or more additional PAH medications (Table 1). Based on self-report, most current users (10/15) had been treated with selexipag for 12 months or more; three current users had been taking selexipag for 6 to 12 months, and two for less than 6 months. Three of the 15 (20%) participants currently taking selexipag were interviewed during their titration phase, while the remaining 12 were taking a maintenance dose (see Table S1 in Supplementary Information). All five participants not currently taking selexipag had taken selexipag for at least 6 months before discontinuing this medication (Table S1).

### Concept elicitation

**Fig. 1:**
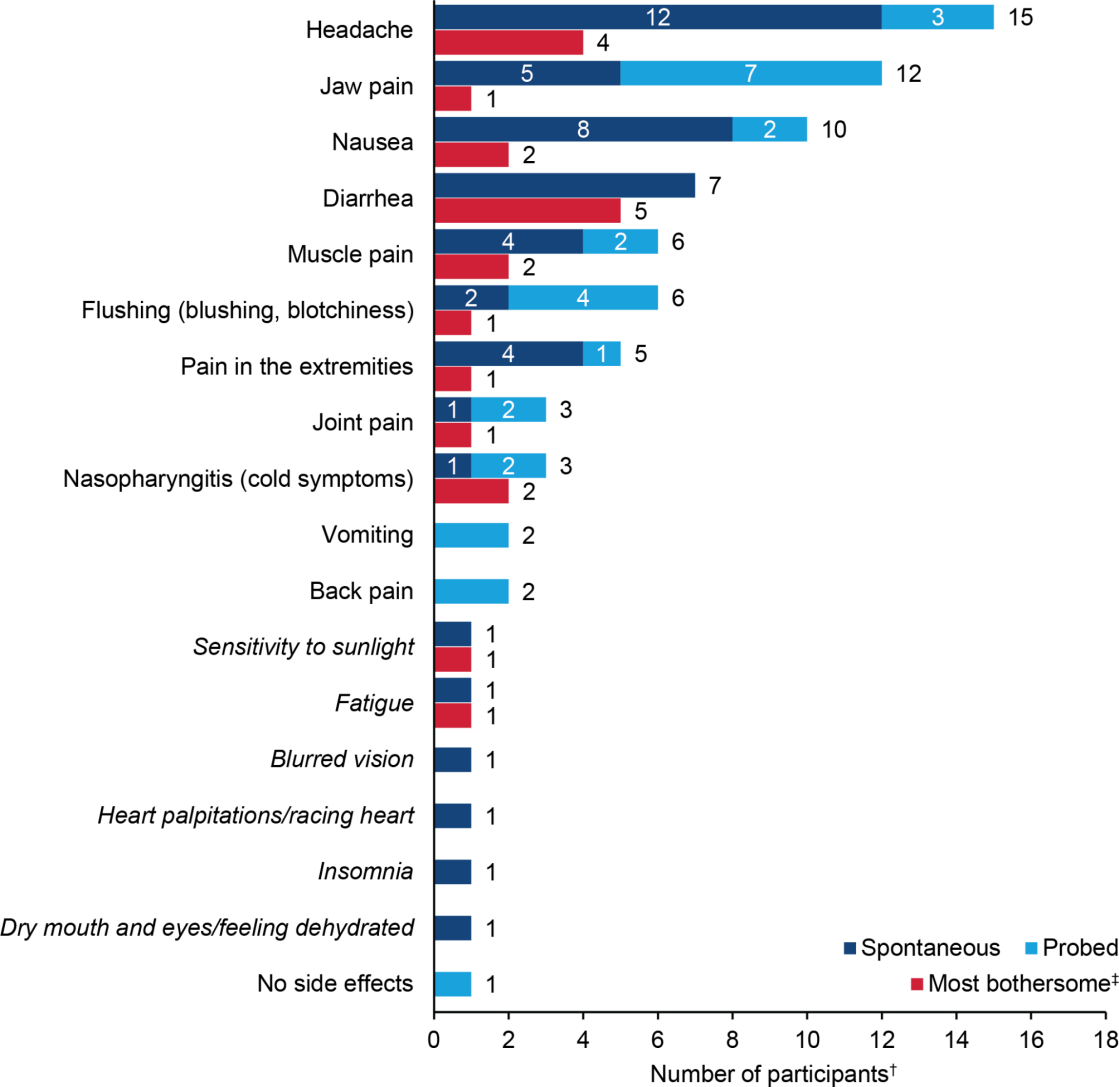
Selexipag AEs reported during concept elicitation (N = 19*) AE: adverse event; PAH: pulmonary arterial hypertension Note: Concepts listed in italics were spontaneously reported by a single participant each, but they were not included in the interview guide and therefore were not probed with all participants *One participant is not included in this figure because she reported experiencing a wide variety of side effects (headache, nasopharyngitis, nausea, flushing, diarrhea, dry heaving, numbness in hand/arm, gas/belching) but stated there was no way she could determine the cause of any of them because she had been on three medications simultaneously for the duration of her PAH treatment ^†^Included in these counts are participants who reported experiencing the following side effects but are unsure whether they were caused by selexipag: headache (3), jaw pain (2), back pain (2), nasopharyngitis (2), flushing (1), vomiting (1), blurred vision (1), dry mouth and eyes/feeling dehydrated (1) ^‡^One participant reported low oxygen as the most bothersome side effect but later attributed the low oxygen to unmanaged PAH. Most bothersome side effects for the remaining 17 participants are shown, but counts do not sum to 17 because participants could select more than one most bothersome side effect

Symptomatic AEs reported by participants are presented in Fig. 1. The most frequently reported AEs—all of which were included among the items selected for debriefing—were headache, jaw pain, and nausea, reported by 15, 12, and 10 participants, respectively. There was no clear pattern as to which AEs were the most bothersome, with 11 different AEs identified as being most bothersome across the 20 interviews. Diarrhea and headache were identified as the most bothersome symptoms by 5 and 4 participants, respectively. Nausea, muscle pain, and nasopharyngitis were each reported to be the most bothersome symptom by 2 participants.

The timing of when participants recalled experiencing AEs is presented in Table 2. No clear pattern for timing of AEs emerged—notably, the number of participants who reported experiencing an AE during the maintenance phase of selexipag treatment was not always lower than at treatment initiation or during the titration phase.

**Table 2:** Timing of side effects reported by participants (N = 19*)

AE	N	When experienced^ †^ (n)		
		At treatment initiation	During titration	Maintenance
Headache	15	7	11	7
Jaw pain	12	5	7	9
Nausea	10	4	5	5
Diarrhea	7	4	3	5
Muscle pain	6	2	4	5
Flushing (blushing, blotchiness)	6	4	2	3
Pain in the extremities	5	4	5	1
Joint pain	3	0	1	1
Nasopharyngitis (cold symptoms)	3	3	0	1
Vomiting	2	0	1	1
Back pain	2	0	0	1
*Sensitivity to sunlight*	1	0	0	1
*Fatigue*	1	1	1	0
*Heart palpitations/ racing heart*	1	0	1	0
*Insomnia*	1	0	0	1
*Dry mouth and eyes/ feeling dehydrated*	1	1	1	1

AE: adverse event; PAH: pulmonary arterial hypertension

Note: In some cases, participants reported experiencing a side effect during multiple periods or failed to answer a follow-up question, so counts in each section do not always sum to the number reporting a given side effect. Concepts listed in italics were spontaneously reported by a single participant each, but they were not included in the interview guide and therefore were not probed with all participants

*One participant is not included in this table because she reported experiencing a wide variety of side effects (headache, nasopharyngitis, nausea, flushing, diarrhea, dry heaving, numbness in hand/arm, gas/belching) but stated there was no way she could determine the cause of any of them because she had been on three medications simultaneously for the duration of her PAH treatment

^†^“At treatment initiation” represents reports of the side effect starting right away or very soon after initiating treatment with selexipag. “During titration” includes participants who reported the side effect started at some point during the titration period. This includes participants for whom the side effect continued steadily after it began, and those for whom the side effect started or worsened with each dosage increase and leveled out until the next increase. “Maintenance” includes participants who reported experiencing the side effect when they were on a stable maintenance dosage of selexipag

Examples of participant responses about selexipag AEs in their own words are presented in Table 3. Among participants who reported experiencing a given AE, there was variability in the severity of that AE, as can readily be seen by comparing the selected quotes regarding headache. Interviews revealed that some AEs were transitory and had specific triggers (e.g., quote for jaw pain), whereas others were long-lasting (e.g., quote for muscle pain). Interviews also revealed that some participants reported an AE was associated with up-titration but subsided during the maintenance phase (e.g., quote for pain in the extremities). The only AE for which a consistent pattern emerged was jaw pain, which was reported to lessen in severity and frequency but remain present at maintenance dosage for participants who experienced it.

**Table 3:** Example responses during concept elicitation, in patients’ own words

AE/theme	Response
Headache	I got there [to maintenance], after 2 weeks then they finally subsided and I just, I told my doctor that was my mercy cry. I just could not force myself to do it again. I was just done. The headaches were debilitating. I couldn’t function. I couldn’t think straight. I just didn’t want to do anything. And then when you add in the extremity pain, it was just… you were this big ball of pain that just, you just didn’t want to do anything but try to zone out and do the best you could to cope. It took all my energy to cope with the side effects. (Patient 2)
	Super mild. It’s also associated with while I’m at work […] But yeah, very tolerable with Tylenol. More of like…I would be able to tolerate it without Tylenol. I just need my brain to be at its best while I’m working, so I just take it anyway. (Patient 12)
Jaw pain	That usually comes with the first bite or drink of something and it’s real sharp and it hurts but once it fades down, it goes away. And it usually only happens that first bite. […] When it’s happening, you think it’s severe because it hurts that bad. But it only lasts for a few seconds really, so I think it’s more bothersome than severe. I mean, it’s not by any means, but it is what it is and it doesn’t last. I can tolerate that. (Patient 20)
Nausea	That seemed to start right when I started the medication feeling kind of sick to my stomach, and I think it happened after the first titration, I felt it a little bit more. […] And then I went up and the medication again and it felt a little bit more nauseated. I wasn’t throwing up, but just feeling like overall yuck. And like it subsided after a few months. (Patient 17)
Diarrhea	When it hits when I’m about to go out, it makes me late. And then I got to find a toilet while I’m out in the street. And it’s kind of embarrassing being in the bathroom and stinking it up. And they know it’s you, because it’s only 2 stalls or something like that, or it’s one stall, and if somebody’s trying to get in that 1 stall but you’re there stuck. It’s going out in public and actually have to go and race to toilets, so you learn where the toilets and the clean bathrooms are with this disease. You can’t be one of them people that don’t use public washrooms or you’ll be stuck in the house. (Patient 4)
Muscle pain	I started noticing more muscle pain right after I started doing the titration, I guess. Just sore, achy muscles. My calves. […] It was pretty constant, the achy muscles and joints during this whole process, and I still have achy muscles and joints. And there’s…sometimes I’d get muscle cramps in my calves, sometimes my hamstring. I get a muscle cramp. […] More severe during titration than now. It’s just nagging muscle aches now. […] It’s not an everyday thing. It’s more of a nagging. Like everything else, just a nagging. If it gets really sore, I’ll just take some Tylenol. (Patient 1)
Flushing	It’s just like, I feel like I’ll get red in the face or my arms mostly and sometimes in my chest. Like hot, it feels hot… and I start sweating. It’s just uncomfortable. (Patient 18)
Pain in the extremities	I think every time I’d titrate up it was 1 or 2 days of some leg pain and so I’d put my legs up for maybe 20 min and then I was fine. After those 2 days, there was nothing. (Patient 5)
Joint pain	Sometimes I’ll just wake up in the middle of the night and all of my major joints—my shoulders, my hips, elbows, everything—all my major joints are…I have to take Tramadol. I don’t take medication for fun but I have to take an actual opioid, which I don’t love, because it’s that painful and Tylenol doesn’t…PAH patients can’t take NSAIDs, so it’s Tylenol or an opioid pain medication. So that sucks. (Patient 7)
Nasopharyngitis	So at first, you’re very stuffy. It’s hard to explain that. But it’s like you have a really bad head cold, without any of the…snot, I guess is the not-so-nice way to say that. To the point that I remember having to get up in the middle of the night and go just sit in a recliner and try to sleep that way. Because I just couldn’t breathe. (Patient 3)
Pretreatment discussion with healthcare provider	My doctor talked about possible headaches, leg pain, nausea, throwing up, possible diarrhea, all of these different things. (Patient 5)

AE: adverse event; GI: gastrointestinal; NSAID: nonsteroidal anti-inflammatory drug; PAH: pulmonary arterial hypertension

### Cognitive debriefing

#### Comprehension of items and response scales

During the cognitive debriefing portion of the interviews, all participants reported that they would have no difficulty recalling the AEs they experienced in the previous 7 days, and that they would be able to answer each of the questions easily and accurately in a clinical trial setting.

#### Relevance of PRO-CTCAE items

The numbers of participants who endorsed each PRO-CTCAE item as relevant to capture in a PAH clinical trial are shown in Table 4. All of the questionnaire concepts were endorsed as relevant by nearly all participants asked.

**Table 4:** Perceived relevance of PRO-CTCAE items (N = 20)

PRO-CTCAE item	No. of participants debriefed on item	Attributes measured*	Relevant	Not relevant	Participants who had personally experienced (N = 19)
Round 1 and Round 2 interviews					
Headache	20	F, S, I	20	0	15
Nausea	20	F, S	20	0	10
Loose or watery stools (diarrhea)	20	F, I^ †^	18	2	7
Aching muscles	20	F, S, I	18	2	6
Aching joints	20	F, S, I	17	3	3
Vomiting	20	F, S	17	3	2
Round 1 interviews only					
Pain	10	F, S, I	9	1	5^ ‡^
Cough	10	S, I	7	3	0
Round 2 interviews only					
Jaw pain	10	F, S, I	8	2	12^ §^
Blushing	10	F, S	9	1	6

PRO-CTCAE: Patient-Reported Outcomes version of the Common Terminology Criteria for Adverse Events

*F = frequency; S = severity; I = interference with daily life

^†^The interference item was added for the final 10 (Round 2) interviews

^‡^Five participants reported “pain in the extremities,” but the PRO-CTCAE item is a general pain item

^§^Twelve participants reported jaw pain during concept elicitation, but this item was only presented as an item for debriefing following midpoint revisions, in the final 10 interviews

Because none of the first 10 participants reported experiencing cough as a side effect of selexipag, and cough can be a symptom of PAH, it was decided to delete cough from the list of items for the Round 2 interviews and add a new item on blushing (flushing). The majority of the 10 Round 2 participants reported that “flushing” was a more appropriate and more recognized word than “blushing”, which some participants associated with being embarrassed rather than a side effect of medication.

When asked what came to mind upon reading the general pain item (presented before those addressing specific types of pain), most Round 1 interview participants mentioned jaw pain, muscle pain, joint pain, pain in the extremities, and/or headache. As these Round 1 interviews indicated the PRO-CTCAE general pain item was of limited value, for Round 2 this item was modified to ask specifically about jaw pain. When queried, none of the Round 2 participants reported experiencing any type of pain (as a side effect of oral selexipag) that was not captured in the questionnaire.

Other than blushing/flushing and jaw pain (which were added for the Round 2 interviews), nasopharyngitis (n = 3) was the only medication-associated AE experienced or mentioned as missing from the PRO-CTCAE items by more than one participant.

Based on these interviews, the following items were retained and would be relevant to include in a PRO questionnaire to assess tolerability of a medication targeting the prostacyclin pathway in a PAH clinical trial: nausea, vomiting, diarrhea, flushing, jaw pain, headache, aching muscles, and aching joints.

#### Dimensions for assessing PRO-CTCAE items

When asked whether frequency, severity, and/or interference seemed appropriate to capture for each AE, participants generally endorsed all of these aspects. In the Round 2 interviews, three of the 10 participants commented that interference might not be needed for jaw pain given the fleeting nature of this side effect.

#### Frequency of questionnaire assessment

When asked how often clinical trial participants should be asked to complete the questionnaire during the titration period in order to capture a complete picture of the patient experience, interview participants were nearly unanimous in stating that it should be weekly. When asked what frequency is appropriate during the maintenance period, the most common recommendation was for monthly administration.

## Discussion

Findings from interview participants with PAH support previous research in oncology indicating that the patient perspective on the relevant side effects of treatment is broader than clinician-reported severity and frequency of AEs [39]. Thus, obtaining patients’ reports of drug tolerability should be considered an important addition to clinician-reported assessments.

The present study is one of very few in the literature to use PRO-CTCAE outside oncology [19–21], and to our knowledge is the first to do so in patients with PAH. Items from the PRO-CTCAE library, supplemented by items about additional symptomatic AEs reported in the pivotal selexipag clinical trial, GRIPHON, were judged to be relevant by most of the participants who saw them, even though some of the referenced side effects were only experienced by a few participants. Frequent and bothersome side effects of selexipag, such as pain and gastrointestinal AEs, are well recognized in current clinical practice, and patients are often proactively prescribed antiemetics and/or mild analgesics to manage expected side effects during the titration phase [27, 40].

Although the side effects reported to be associated with selexipag in this study were aligned with the previously described AE profile attributable to the mechanism of action of this drug [28], these interviews revealed a high degree of heterogeneity among participants in how they experience these AEs in terms of frequency, severity, time of onset, and changes in frequency and severity over the course of titration and into the maintenance phase. There was no clear pattern in which AEs were the most bothersome. These insights will be important to consider in future studies of medications targeting the prostacyclin pathway to ensure that the full range of patient experience is adequately captured.

PRO tools are among several measures advocated in expert recommendations for PAH trial design to capture patient-relevant aspects of treatment efficacy and safety [41, 42]. FDA guidance on how the agency evaluates PRO instruments to support claims in medical product labeling notes the need to measure the adverse consequences of treatment separately from the effectiveness of treatment [43]. Capturing the patient perspective on tolerability can provide a more complete understanding of the overall treatment experience of patients receiving a drug. The final items selected in this study may be useful to assess tolerability of treatments targeting the prostacyclin pathway.

This study had several strengths, including item selection based on identification of AEs relevant to patients taking selexipag from literature review and patient self-report, and the inclusion not only of current users of selexipag but also of patients no longer taking the drug. Participant characteristics were generally representative of patients with PAH currently seen in routine clinical practice in the US in terms of average age and gender distribution, based on recent data from the United States Pulmonary Hypertension Scientific Registry [44]. The recruitment of only one participant with severe PAH in WHO FC IV reflects the fact that selexipag is indicated for patients in WHO FC II–III [29, 30]. Patients in WHO FC IV would be expected to be prescribed a parenteral prostanoid, rather than an oral medication targeting the prostacyclin pathway such as selexipag, as recommended in US and international clinical practice guidelines available during the study period [45–48] and the most recent update of European guidelines [22]. The study followed the recommended process of content validation and item selection from the PRO-CTCAE library [13].

The study also had limitations. Questions were selected based on the side-effect profile of oral selexipag and were not intended to be comprehensive of all medications targeting the prostacyclin pathway, and thus could not cover AEs that may be intrinsic to other drugs’ pharmacology and/or their different routes of administration. Specifically, although the common side effects of selexipag related to its pharmacological action are shared with other prostacyclin-pathway agents, oral selexipag avoids the risk for catheter-related AEs associated with continuous intravenous infusion, infusion-site AEs associated with continuous subcutaneous infusion, and cough and throat irritation associated with inhaled medications [40]. These additional AEs would need to be considered when designing assessments of tolerability for intravenous, subcutaneous, or inhaled medications.

As all participants were in the US and English-speaking, findings may not be generalizable to patients in other countries or speakers of other languages. Future research in this field should strive to oversample racial and ethnic minorities to obtain more representative study populations.

Furthermore, PAH diagnoses were self-reported by participants and not independently verified (e.g., via chart review or follow-up with treating clinicians).

## Conclusions

Insights from this qualitative research in patients with experience taking selexipag for PAH support the importance of using PRO instruments to measure tolerability during drug development, and the applicability of PRO-CTCAE items outside of oncology. The PRO-CTCAE items selected in this study and the additional symptomatic AEs identified as patient-relevant have the potential to inform tolerability in future studies of selexipag and possibly other PAH therapies.

### Electronic supplementary material

Below is the link to the electronic supplementary material.

Supplementary Material 1

Supplementary Material 2

Supplementary Material 3

Supplementary Material 4

## Data Availability

The qualitative data in this study (transcripts and interview notes) will not be made publicly available as they contain information that could compromise research participant consent.

## Abbreviations

AE: adverse event
EMA: European Medicines Agency
FC: functional class
FDA: Food and Drug Administration
GED: General Educational Development
GI: gastrointestinal
NCI: National Cancer Institute
NSAID: nonsteroidal anti-inflammatory drug
PAH: pulmonary arterial hypertension
PH: pulmonary hypertension
PRO: patient-reported outcomes
PRO-CTCAE: Patient-Reported Outcomes version of the Common Terminology Criteria for Adverse Events
RA: rheumatoid arthritis
SD: standard deviation
WHO: World Health Organization
