# Supplementary material for: Use of the National Cancer Institute Patient-Reported Outcomes version of the Common Terminology Criteria for Adverse Events to assess treatment tolerability in pulmonary arterial hypertension: qualitative patient research findings in current and former users of oral selexipag
Source: J Patient Rep Outcomes. 2023 Dec 18;7:134. doi: 10.1186/s41687-023-00673-w (PMC10728389; doi:10.1186/s41687-023-00673-w)
Supplement: Supplementary file 1 — Supplementary Material 1 [file 41687_2023_673_MOESM1_ESM.docx]

# Supplementary Information

## Additional file 1

Table S1. Time treated with selexipag by current status

| Time treated with selexipag | Selexipag status | | |
| --- | --- | --- | --- |
|  | Currently taking  (n = 15) | Discontinued  (n = 5) | Total  (N = 20) |
| Less than 6 months | 2 | 0 | 2 |
| 6 months up to 12 months | 3 | 2 | 5 |
| 12 months up to 24 months | 2 | 1 | 3 |
| 24 months or more | 8 | 2 | 10 |

Note: Among participants currently taking selexipag, both of those who had begun less than 6 months ago and 1 participant who began 6 to 12 months ago reported still titrating at the time of the interview.
