# Supplementary material for: Use of the National Cancer Institute Patient-Reported Outcomes version of the Common Terminology Criteria for Adverse Events to assess treatment tolerability in pulmonary arterial hypertension: qualitative patient research findings in current and former users of oral selexipag
Source: J Patient Rep Outcomes. 2023 Dec 18;7:134. doi: 10.1186/s41687-023-00673-w (PMC10728389; doi:10.1186/s41687-023-00673-w)
Supplement: Supplementary file 3 — Supplementary Material 3 [file 41687_2023_673_MOESM3_ESM.docx]

# JPRO-D-23-00182: Responses to peer reviewer comments

| **Peer reviewer comment** | **Response** | **Change in manuscript** |
| --- | --- | --- |
| **Reviewer #1** |  |  |
| 1. I congratulate the authors on a well-written manuscript describing a study supporting rigorous characterization of patient experiences of symptomatic adverse events beyond oncology. The manuscript is clearly written and provides well considered background and discussion of the value of and methods to consider tolerability in drug development using the PRO-CTCAE (including appropriate modifications) beyond the original application to quantify tolerability associated with oncology treatments. I have two issues for the authors to consider for revision. | The authors thank Reviewer 1 for this positive appraisal. | N/A |
| 1. There is no rationale provided for inclusion of 20 patient interviews split between 15 currently on therapy and 5 who discontinued in the past 12 months. There is no concept saturation for the CE portion of the interviews to indicate that all relevant concepts were identified. Please add rationale for the sample size and decision to not conduct an analysis of concept saturation. | The sample size of 20 was planned based on previous research indicating that saturation can be reached in as few as 10–12 interviews [1-3].  The split between on- and off-selexipag patients was not prespecified, but simply happened to be the distribution in the recruited sample, given the eligibility criteria described in the Methods: patients currently taking selexipag or stopped within the past 12 months. We knew that the titration process can be challenging for some patients, leading them to stop selexipag therapy during this period [4]. As we wanted to ensure we did not exclude anyone who could speak to the tolerability of selexipag, we opted to extend the eligibility criteria to anyone who recently discontinued the drug. We thus achieved a mixed sample of patients, comprising those who overcame the titration process successfully, as well as others who did not and decided to stop. Because we were uncertain how many patients we would be able to recruit in the two categories, we did not set hard targets for the proportion on vs. off selexipag.  Given the previously mentioned findings of prior research on concept saturation, the nature of the present study questions, and the well-characterized AE profile of selexipag and other prostacyclin-pathway therapies in the literature, it was not considered necessary to conduct a formal analysis of concept saturation. | Methods>Participants, p 5: Using an in-house patient database, Rare Patient Voice identified potentially eligible participants. Individuals who expressed interest in participating in the study were screened for eligibility and those who qualified were scheduled for interviews. A sample size of 20 was planned based on previous research indicating that concept saturation can be reached in as few as 10–12 interviews [1-3].  Methods>Participants, pp 5–6: The following eligibility criteria were applied: … Including participants who had recently discontinued selexipag ensured that important views on the tolerability of the drug were captured, given that the titration process is based on tolerability and therefore can be challenging, leading to some patients stopping selexipag therapy during this period [4]. |
| 1. As the authors noted with references by Stanulovic (2) and Basch (3), the terminology and meaning of terms for tolerability, adverse and related concepts are not well established and these terms are often used in conflicting ways. However, the term Adverse Event is a strictly defined by the FDA, has defined regulatory standards and may include both symptomatic events as well as physiologic and laboratory events. This paper focuses on symptomatic tolerability or symptomatic adverse events. Please consider clarifying adverse events as "symptomatic adverse events" or clearly defining the terminology as used throughout the manuscript. | The authors thank Reviewer 1 for this helpful point regarding appropriate terminology. We have added “symptomatic” at appropriate locations throughout the manuscript to clarify that we are referring to symptomatic AEs. | We added “symptomatic” before “AEs” in the following locations:   - Abstract:   - Methods, p 1 [first occurrence only]   - Conclusions, p 2 - Background, p 4 - Methods:   - Study design, p 5   - Concept elicitation, p 7 [first occurrence only] - Results:   - Concept elicitation, p 9 [first occurrence only] - Discussion, p 11 [second paragraph only] - Conclusions, p 13 |
| **Reviewer #2** |  |  |
| 1. This is a well written article and interesting research. However, it relates specifically to oral selexipag, the authors should make that clear in title and background within abstract and main body. | The authors thank Reviewer 2 for this positive evaluation. We agree with the helpful advice to clarify that the study relates specifically to oral selexipag, and have added “selexipag” in the manuscript title, Abstract Background, and main body Background as suggested. | Title Page, p 1: Use of the National Cancer Institute Patient-Reported Outcomes version of the Common Terminology Criteria for Adverse Events to assess treatment tolerability in pulmonary arterial hypertension: qualitative patient research findings in current and former users of oral selexipag  Abstract>Background, p 1: This qualitative interview study assessed the suitability of items selected from the PRO-CTCAE library for assessing tolerability of selexipag, a medication targeting the prostacyclin pathway for patients with pulmonary arterial hypertension (PAH).  Background, p 5: Specifically, qualitative interviews were conducted in patients with PAH and prior experience with selexipag to inform the development of a PRO measurement strategy for implementation in clinical trials evaluating and comparing the tolerability of selexipag and potentially other medications targeting the prostacyclin pathway. |
| 1. Make it clear in abstract that it was 20 interviews in total (10 in each) | We have clarified that there were two rounds of 10 interviews in the Abstract Methods. Between this addition and the mention of 20 participants at the start of the Abstract Results, the reader will be clear that there were 20 interviews in total. | Abstract>Methods, p 1: Two rounds of 10 qualitative, web-assisted telephone interviews following a semi-structured guide were conducted in individuals with recent experience taking oral selexipag for PAH. |
| 1. In the intro the authors argue that the desire to adhere to dose or intensity of treatment should be assessed. That is not reported, was this question asked in the interviews? | We acknowledge that this question was not asked in the interviews, so we have deleted mention of this in the Background section to avoid setting the reader’s expectation that results for this question will be reported. | Background, p 3: To provide a more complete picture of the patient experience and support informed therapeutic decision-making, assessment of tolerability should incorporate direct measurement of the patient’s feelings and functioning [6]. |
| 1. It is unclear why authors bring in the notion of focusing on the subgroup of PH in PAH when they then continue to discuss PAH treatments and refer to PAH in objectives. | PAH is a subgroup of PH, so the reason we mention PH in the Background section is to orient readers to the category of diseases to which PAH belongs. We have revised the manuscript title to clarify that the study was in patients with PAH, not PH generally, in alignment with the study’s eligibility criteria. | Title Page, p 1: Use of the National Cancer Institute Patient-Reported Outcomes version of the Common Terminology Criteria for Adverse Events to assess treatment tolerability in pulmonary arterial hypertension: qualitative patient research findings in current and former users of oral selexipag |
| 1. Is the RTI international ethics board appropriate to review or should it be independent of the company doing the research? | The authors thank Reviewer 2 for raising this question. Indeed, we consider that this is appropriate since RTI holds a Federal-Wide Assurance (FWA #3331 effective until January 26, 2026) from the Department of Health and Human Services (DHHS) Office for Human Research Protections (OHRP) that allows RTI to review and approve human subjects protocols through their IRB committees. These committees are also registered with OHRP for both DHHS and FDA-regulated research (registration expires January 20, 2025). The FWA requires IRB review for all studies conducted by RTI that involve human subjects, regardless of the funding source.  While we feel that adding this level of additional detail to the manuscript may not be warranted, we are grateful for this opportunity to address the reviewer’s concern. | N/A |
| 1. The authors should make it clear in the article title, objectives, and main text, that this is specific to selexipag, and should provide more considerations about translating the findings from just selexipag to other meds targeting the prostacyclin pathway in the discussion. Are there any limitations in focusing only on one treatment in this study? Discuss potential idiosyncrasies of selexipag and generalizability of results to other treatments as appropriate. | As noted in our response to comment 1 from Reviewer 2, we agree with the helpful suggesting to clarify that the study relates specifically to oral selexipag, and have added “selexipag” in the manuscript title, Abstract Background, and main body Background as suggested.  Regarding the reviewer’s request to provide more details on how these findings for selexipag may apply (or not) to other drugs targeting the prostacyclin pathway, we have added a clarification in the manuscript Discussion that the AEs of selexipag related to its pharmacological action are all observed in other medications targeting the prostacyclin pathway, and have supported this statement with addition of a review article on these AEs [7].  We also note that there are administration-route–related differences in the AE profile of oral selexipag compared with prostacyclin-pathway agents administered by continuous IV infusion via implanted catheter, SC infusion, or inhalation [7], and we have added this limitation to the generalizability of this study in the Discussion | Title Page, p 1: Use of the National Cancer Institute Patient-Reported Outcomes version of the Common Terminology Criteria for Adverse Events to assess treatment tolerability in pulmonary arterial hypertension: qualitative patient research findings in current and former users of oral selexipag  Abstract>Background, p 1: This qualitative interview study assessed the suitability of items selected from the PRO-CTCAE library for assessing tolerability of selexipag, a medication targeting the prostacyclin pathway for patients with pulmonary arterial hypertension (PAH).  Background, p 5: Specifically, qualitative interviews were conducted in patients with PAH and prior experience with selexipag to inform the development of a PRO measurement strategy for implementation in clinical trials evaluating and comparing the tolerability of selexipag and potentially other medications targeting the prostacyclin pathway.  Discussion, pp 12–13: Questions were selected based on the side-effect profile of oral selexipag and were not intended to be comprehensive of all medications targeting the prostacyclin pathway, and thus could not cover AEs that may be intrinsic to other drugs’ pharmacology and/or their different routes of administration. Specifically, although the common side effects of selexipag related to its pharmacological action are shared with other prostacyclin-pathway agents, oral selexipag avoids the risk for catheter-related AEs associated with continuous intravenous infusion, infusion-site AEs associated with continuous subcutaneous infusion, and cough and throat irritation associated with inhaled medications [7]. These additional AEs would need to be considered when designing assessments of tolerability for intravenous, subcutaneous, or inhaled medications. |
| 1. I'm not quite sure that the sample can be considered diverse in race/ethnicity as claimed in the results section. All US, English speaking only. Hispanic is a fairly large proportion of US population and not proportionally represented. This should be discussed more in the limitations section. | We have reworded the Abstract and Results sections to avoid stating that the sample is “diverse in race/ethnicity”.  The limitations paragraph in the Discussion highlighted that this US study may not be generalizable to other countries, to which we have added the limitations around language and representation of racial and ethnic minorities as suggested by Reviewer 2. | Abstract>Results, p 1: Interviews were conducted with 20 participants with PAH (mean [range] age 50 [24–68] years; 75% female; 85% in World Health Organization Functional Class II–III), comprising different races/ethnicities, levels of education, and employment status.  Results>Participant characteristics, p 8: As presented in Table 1, participants ranged in age from 24 to 68 years (mean 50 years), were predominantly (75%) female, comprised different races/ethnicities, and were diverse in level of education and employment status.  Discussion, p 13: As all participants were in the US and English-speaking, findings may not be generalizable to patients in other countries or speakers of other languages. Future research in this field should strive to oversample racial and ethnic minorities to obtain more representative study populations. |
| 1. How long had those currently taking selexipag taken it for? This information should be added to the main text easily. | We have added a description of the duration of selexipag use in the study sample to the Results section. | Results>Participant characteristics, pp 8–9: Nearly all participants currently taking oral selexipag (14 of 15; 93%) were taking two or more additional PAH medications (Table 1). Based on self-report, most current users (10/15) had been treated with selexipag for 12 months or more; three current users had been taking selexipag for 6 to 12 months, and two for less than 6 months. Three of the 15 (20%) participants currently taking selexipag were interviewed during their titration phase, while the remaining 12 were taking a maintenance dose (see Table S1 in Supplementary Information). All five participants not currently taking selexipag had taken selexipag for at least 6 months before discontinuing this medication (Table S1). |
| 1. In the CE results (line 187-191). Make it more explicit that there was no clear pattern on which was the most bothersome AE - out of 20 interviews 11 different AEs were identified as being most bothersome by somebody. Headache and diarrhea were most commonly identified as most bothersome, but out of 15 reporting headache, it was only most bothersome for 4. With the exception of nausea, those most frequently experienced were only reported as most bothersome to a few of those experiencing it. This pattern across the AE responses should be discussed by the authors. | The authors thank the reviewer for this helpful suggestion and have added these points to the CE results paragraph as suggested. | Results>Concept elicitation, p 9: The most frequently reported AEs—all of which were included among the items selected for debriefing—were headache, jaw pain, and nausea, reported by 15, 12, and 10 participants, respectively. There was no clear pattern as to which AEs were the most bothersome, with 11 different AEs identified as being most bothersome across the 20 interviews. Diarrhea and headache were identified as the most bothersome symptoms by 5 and 4 participants, respectively. Nausea, muscle pain, and nasopharyngitis were each reported to be the most bothersome symptom by 2 participants.  Discussion, p 12: Although the side effects reported to be associated with selexipag in this study were aligned with the previously described AE profile attributable to the mechanism of action of this drug [8], these interviews revealed a high degree of heterogeneity among participants in how they experience these AEs in terms of frequency, severity, time of onset, and changes in frequency and severity over the course of titration and into the maintenance phase. There was no clear pattern in which AEs were the most bothersome. These insights will be important to consider in future studies of medications targeting the prostacyclin pathway to ensure that the full range of patient experience is adequately captured. |
| 1. It would also be interested to explore whether there were any patterns in the relationship between experiencing a symptom and how bothersome it was? | As noted in our response to comment 9 from Reviewer 2,  there was no clear relationship observed. We have added this point to the Results and Discussion sections. | Please see manuscript changes identified in the previous row for comment 9. |
| 1. What was the data on frequency, severity and interference associated with each AE? This should be presented, and also considered by the authors in understanding which AEs are most bothersome and why. | The authors appreciate the reviewer’s request, and indeed had carefully considered the suitability of these data for inclusion when drafting the manuscript. While we asked participants which aspect of AEs was most important, in practice patients often have a difficult time distinguishing between frequency and severity as they are often coupled (i.e., patients frequently consider a symptom to be more severe if they experience it more of the time). Given this consideration and the relatively small sample size, we felt these data should be interpreted with caution and would not strengthen the paper. Indeed, comparing these aspects of AEs was not a focus of the study or this paper—instead, our main goal was to identify which aspects of each symptom patients thought were important for assessment in a clinical trial. We recommend assessing each aspect that is relevant for each side effect in future studies with adequate sample sizes.  Instead of including these weak data from the study in the manuscript, we have added in the Discussion section a new sentence describing how the known frequent/bothersome AEs of oral selexipag are dealt with proactively in clinical practice. | Discussion, p 11: Items from the PRO-CTCAE library, supplemented by items about additional symptomatic AEs reported in the pivotal selexipag clinical trial, GRIPHON, were judged to be relevant by most of the participants who saw them, even though some of the referenced side effects were only experienced by a few participants. Frequent and bothersome side effects of selexipag, such as pain and gastrointestinal AEs, are well recognized in current clinical practice, and patients are often proactively prescribed antiemetics and/or mild analgesics to manage expected side effects during the titration phase [7, 9]. |
| 1. Why is the 1 participant who could not attribute AEs to selexipag not included, but the counts include data from others who could not attribute the AE to selexipag? On the basis of excluding the 1 participant, should not data on any AE that cannot be attributed to selexipag also be excluded? | We believe that the reviewer is referring to the apparent discrepancy between:   - (A) exclusion of the one participant who reported experiencing a wide variety of side effects (headache, nasopharyngitis, nausea, flushing, diarrhea, dry heaving, numbness in hand/arm, gas/belching) but stated there was no way she could determine the cause of any of them because she had been on three medications simultaneously for the duration of her PAH treatment and - (B) inclusion in the counts of participants who experienced the following side effects but were unsure whether they were caused by oral selexipag: headache (3), jaw pain (2), back pain (2), nasopharyngitis (2), flushing (1), vomiting (1), blurred vision (1), dry mouth and eyes/feeling dehydrated (1).   The reason for this different approach is because participant A stated explicitly that there was no way she could make attributions of any AE to selexipag vs. other medications, whereas the (B) participants judged that the side effects noted were likely attributable to selexipag based on timing or other factors; they just were not entirely certain. | N/A |
| 1. If participants could select more than one most bothersome AE, they are not the "most" bothersome, but bothersome. Can the authors include the way that this question was asked to clarify? Did the interviewer ask why something was identified as most bothersome? | The way the question was posed did not call for participants to identify the single most-bothersome side effect but rather to identify which of the side effects were most bothersome. We have added the exact questionnaire wording in the Methods to clarify this point. We can confirm that the interviewer did ask why the most-bothersome side effects were so identified, and this has also been added in the Methods. | Methods>Concept elicitation, p 7: Interview participants were asked to describe all symptomatic AEs they experienced when taking selexipag, along with the frequency and severity of each side effect, the stage of treatment (i.e., titration or maintenance) at which it was experienced, any changes in either frequency or severity that they experienced over time, and whether and how the side effect interfered with their daily life. Participants were also asked, “Thinking about any negative effects that you experienced, which were the most bothersome to you? Why did these bother you the most?” |

# References

1. Francis JJ, Johnston M, Robertson C, Glidewell L, Entwistle V, Eccles MP, et al (2010) What is an adequate sample size? Operationalising data saturation for theory-based interview studies. Psychol Health. 25(10):1229-45. doi:10.1080/08870440903194015.

2. Vasileiou K, Barnett J, Thorpe S, Young T (2018) Characterising and justifying sample size sufficiency in interview-based studies: systematic analysis of qualitative health research over a 15-year period. BMC Med Res Methodol. 18(1):148. doi:10.1186/s12874-018-0594-7.

3. Turner-Bowker DM, Lamoureux RE, Stokes J, Litcher-Kelly L, Galipeau N, Yaworsky A, et al (2018) Informing a priori sample size estimation in qualitative concept elicitation interview studies for clinical outcome assessment instrument development. Value Health. 21(7):839-842. doi:10.1016/j.jval.2017.11.014.

4. McLaughlin V, Farber HW, Highland KB, Hemnes AR, Chakinala MM, Chin KM, et al (2023) Disease characteristics, treatments, and outcomes of patients with pulmonary arterial hypertension treated with selexipag in real-world settings from the SPHERE registry (SelexiPag: tHe usErs dRug rEgistry). J Heart Lung Transplant. doi:10.1016/j.healun.2023.09.016.

6. Basch E, Campbell A, Hudgens S, Jones L, King-Kallimanis B, Kluetz P, et al (2018) Broadening the definition of tolerability in cancer clinical trials to better measure the patient experience: a Friends of Cancer Research White Paper. https://friendsofcancerresearch.org/wp-content/uploads/Comparative-Tolerability-Whitepaper_FINAL.pdf. Accessed 24 July 2023.

7. Kingman M, Archer-Chicko C, Bartlett M, Beckmann J, Hohsfield R, Lombardi S (2017) Management of prostacyclin side effects in adult patients with pulmonary arterial hypertension. Pulm Circ. 7(3):598-608. doi:10.1177/2045893217719250.

8. Sitbon O, Channick R, Chin KM, Frey A, Gaine S, Galiè N, et al (2015) Selexipag for the treatment of pulmonary arterial hypertension. N Engl J Med. 373(26):2522-33. doi:10.1056/NEJMoa1503184.

9. Lombardi S, Kingman M, Duncan M, Berngard SC, Fernandes T (2018) Titration of pulmonary arterial hypertension therapeutics: experience-based recommendations. Respir Med. 143(139-146. doi:10.1016/j.rmed.2018.09.002.
