# Supplementary material for: Use of the National Cancer Institute Patient-Reported Outcomes version of the Common Terminology Criteria for Adverse Events to assess treatment tolerability in pulmonary arterial hypertension: qualitative patient research findings in current and former users of oral selexipag
Source: J Patient Rep Outcomes. 2023 Dec 18;7:134. doi: 10.1186/s41687-023-00673-w (PMC10728389; doi:10.1186/s41687-023-00673-w)
Supplement: Supplementary file 4 — Supplementary Material 4 [file 41687_2023_673_MOESM4_ESM.docx]

Use of the National Cancer Institute Patient-Reported Outcomes version of the Common Terminology Criteria for Adverse Events to assess treatment tolerability in pulmonary arterial hypertension: qualitative patient research findings in current and former users of oral selexipag
